# *Leptosphaeria maculans*-*Brassica napus* Battle: A Comparison of Incompatible vs. Compatible Interactions Using Dual RNASeq

**DOI:** 10.3390/ijms23073964

**Published:** 2022-04-02

**Authors:** Kaluhannadige R. E. Padmathilake, Wannakuwattewaduge Gerard Dilantha Fernando

**Affiliations:** Department of Plant Science, University of Manitoba, Winnipeg, MB R3T 2N2, Canada; padmatkr@myumanitoba.ca

**Keywords:** *Rlm7*, *AvrLm7*, transcriptomic study, host–pathogen interaction, *Brassica napus*, *Leptosphaeria maculans*

## Abstract

*Leptosphaeria maculans* causes blackleg disease, which is one of the most destructive diseases of canola (*Brassica napus* L.). Due to the erosion of the current resistance in *B. napus*, it is pivotal to introduce new resistant genotypes to the growers. This study evaluated the potential of *Rlm7* gene as resistance to its corresponding avirulence *AvrLm7* gene is abundant. The *Rlm7* line was inoculated with *L. maculans* isolate with *AvrLm7*; UMAvr7; and the CRISPR/Cas9 knockout *AvrLm7* mutant, umavr7, of the same isolate to cause incompatible and compatible interactions, respectively. Dual RNA-seq showed differential gene expressions in both interactions. High expressions of virulence-related pathogen genes-CAZymes, merops, and effector proteins after 7-dpi in compatible interactions but not in incompatible interaction—confirmed that the pathogen was actively virulent only in compatible interactions. Salicyclic and jasmonic acid biosynthesis and signaling-related genes, defense-related *PR1* gene (GSBRNA2T00150001001), and GSBRNA2T00068522001 in the NLR gene family were upregulated starting as early as 1- and 3-dpi in the incompatible interaction and the high upregulation of those genes after 7-dpi in compatible interactions confirmed the early recognition of the pathogen by the host and control it by early activation of host defense mechanisms in the incompatible interaction.

## 1. Introduction

Canola (*Brassica napus* L.) is the number one cash crop in Canada, contributing CAD 29.9 billion to the national economy annually, including more than 207,000 Canadian jobs and CAD 12 billion in wages [1]. Annual global and Canadian canola production is estimated as 70 MMT and 18.7 MMT, respectively [1,2]. Blackleg, caused by the actinomycete fungus *Leptosphaeria maculans* (Desm.) Ces. et de Not. (anamorph: Phoma lingam (Tode ex Fr.) Desm.) is one of the predominant fatal diseases in canola, which causes losses worth more than CAD 900 million per growing season worldwide [3,4,5,6].

Host genetic resistance is a major management tool that can control blackleg disease. Canola exhibits two types of resistance, major gene (qualitative) resistance and minor gene (quantitative) resistance. Canola expresses the gene-for-gene interaction model [7], but with some exceptions. If a host resistance gene matches with a pathogen’s avirulence gene, the host will initiate a hypersensitive reaction (HR), killing the cells surrounding the infected site and stopping further spreading of the pathogen, which is called an incompatible interaction [8]. In contrast, the diseased interaction is called compatible interaction [9]. To date, 18 major *R* genes have been identified in *Brassica* species: *Rlm1-11*, *RlmS*, *LepR1-4*, *BLMR1*, and *BLMR2* [10,11,12,13,14,15,16], but only two (*LepR3* and *Rlm2*) have been cloned [15,16,17]. Genes *LepR3* and *Rlm2* are the alleles of the same gene and encode leucine-rich repeat receptor-like proteins (LRR-RLP) [15,16]. In *L. maculans*, 16 *Avr* genes have been identified and the genes *AvrLm1*, *AvrLm2*, *AvrLm3*, *AvrLm4-7*, *AvrLm5-9*, *AvrLm6*, *AvrLm10*, and *AvrLm11* have been cloned [18,19,20,21,22,23,24,25,26,27].

*R* gene resistance is not consistent over the years and is constantly challenged with the evolution of new races of *L. maculans*. There were several examples of *R* gene breakdown of canola species due to the selection pressure exerted by continuous use of a single *R* gene over time. The breakdown of *Rlm1* in France and ‘sylvestris’ in Australia can be shown as examples [28,29].

The major *R*-gene used by Canadian farmers, *Rlm3*, has been overcome by the pathogen [30]. *Rlm3* was reported in Canadian canola varieties released from the 1990s onwards. *Rlm1* and ’sylvestris’ resistance breakdown happened within a few years, *Rlm3* resistance was very effective until 2005 [30]. By 2012, the frequency of *AvrLm3* in *L. maculans* population had dropped to 2.7% which was one of the lowest frequencies in the pathogen population in Western Canada [31]. Zhang et al. (2016) explained that asexual pycnidiospores becoming the major source of inoculum in Western Canada might have played a significant role due to extremely cold winters and shorter growing seasons. There is a less chance for pathogen evolution in asexual reproduction. However, breaking down of the current *R* gene explains the necessity of introducing new host *R* genes into the seed market to reduce the selection pressure on host genotypes. Based on the high frequency of *AvrLm7* genotype presence in the pathogen population and stability of the corresponding host genotype *Rlm7* in countries where it has already been introduced, *Rlm7* is the next best candidate for host genotype to be introduced into the canola-host gene pool [32,33].

*L. maculans* is a hemibiotrophic pathogen [4]. Biotrophs assimilate nutrients directly from host cells without killing the cells. On the other hand, necrotrophs break into the host cell and cause cell death before nutrient intake. Among all, hemibiotrophs are the most exciting group that exhibits biotrophic nature at the initial phase and emulates the latter’s necrotrophic characteristics [34]. The underlying mechanisms of the above switching nature during plant hemibiotrophic interactions remains elusive.

Plants use phytohormones for signaling to distinguish among the above-mentioned different types of pathogens and activate appropriate responses [35]. Phytohormones are multifaceted signal molecules that play a significant role in plant immunity programs. Salicylic acid (SA), jasmonic acid (JA), and ethylene (ET) play major roles among them [36,37,38]. In addition, phytohormones strongly link with the pathogens’ trophic nature in the plant system [38]. Studies conducted using transgenic and mutant lines revealed that responses against biotrophic pathogens and hemibiotrophic pathogens are in general regulated by SA [36,39]. In contrast, JA and ET replace that role when it comes against necrotrophs [36,40]. That explains that there is reciprocal inhibition between SA and JA. Previous studies exhibiting SA–JA antagonism support plant defense strategies based on the pathogen trophic nature encountered [36,37,41]. Despite that, the SA–JA relationship is not always antagonistic. There is research evidence for positive regulating of SA and JA. Tamaoki et al. (2013) explained that the defense system activated by both SA and JA signaling interaction during the induction of defense response. Agrawal et al. (2000) observed that the disease-resistant marker rice *PR1b* gene is induced by both SA and JA and suggests that there should be at least a partly shared signal transduction pathway used for signaling of both JA and SA. Tsuda et al. (2009) revealed that synergistic and compensatory relationships exist among SA and JA pathways and are important for optimal resistance to different pathogens.

The basic immune system of plants is triggered by pathogens and related molecules, which are detected by extracellular membrane receptors such as receptor-like kinases (RLKs) and receptor-like proteins (RLPs), referred to as the ‘pathogen-associated molecular pattern (PAMP)-triggered immunity’ (PTI) [42]. Pathogens evolved to implement the next level of virulence by possessing effector proteins to defeat basic plant immunity, PTI. Effectors are pathogen proteins that modulate the plant’s innate immunity and facilitate infection [43]. Consequently, some plants evolved intracellular resistant proteins to recognize some pathogen effectors and are called avirulence proteins at the onset of infection and lead to the next level of immunity called ‘effector-triggered immunity’ (ETI) [44]. Avirulence proteins are pathogenicity proteins that are specifically ‘recognized’ by genotypes of the host plant carrying the corresponding resistance proteins mainly under the nucleotide-binding leucine-rich repeat (NLR) receptor family [45]. ETI triggers HR, causing programed cell death (PCD) surrounding the infected site, and thus limiting further pathogen spread. ETI triggered resistant interaction is known as an incompatible interaction, whereas the interaction causing disease is called compatible interaction in a plant system lacking resistant proteins and/or with a pathogen lacking specific avirulence effectors. During ETI induction of plants, both SA and JA accumulation increases simultaneously [46]. Though the past studies showed the crosstalk between SA and JA, the ETI-associated PCD, which does not trigger the susceptibility to necrotrophs explained the cooperative interplay between SA and JA [47]. In addition, phytohormones showed a higher correlation with the trophic switch of a hemibiotrophic pathogen in the plant system, which is still not fully revealed.

In understanding host–pathogen interactions from both contexts simultaneously, scientists have used dual RNA-seq, which is the most promising high-throughput and highly sensitive technique for genome-wide transcriptional studies. Over the past several years, the RNA-seq technique has been widely used to investigate plant pathosystems, including the canola-blackleg system. Several studies have been studied the host system [2,9] and pathogen system [48] separately. In addition, there were few studies have been studied the whole host-pathosystem by dual RNAseq as well [49].

This study was conducted to follow disease progression through a time-course in the *Rlm7* host genotype, which is the next candidate genotype being introduced into the market. A *L. maculans* isolate with *AvrLm7* was used to create an incompatible interaction with the host, in contrast, the *AvrLm7* gene mutated with the same isolate was used to form a compatible interaction. Both isolates were inoculated in Westar genotype to see their performances in a no-*R*-gene background. Genome-wide transcriptomic studies are important to understanding the molecular and genetic background of a host pathosystem. However, this study focused on understanding the *Rlm7-AvrLm7* pathosystem at a genome-wide transcriptomic level. The most exciting point here is using the *Rlm7* genotype to study both incompatible and compatible interactions, both with and muting *AvrLm7* of the same *L. maculans* isolate, with the host and pathogen backgrounds made the same except the candidate avirulence gene, *AvrLm7*. *AvrLm7* gene mutation was carried out using the CRISPR/Cas9 technique in our lab [50]. The main objective of this detailed transcriptomic study was to have a deeper view of the transcriptional responses mediated by canola *Rlm7* genotype as the host, which would be the next potential candidate to be introduced in the Canadian canola market upon facing challenges with the pathogen *L. maculans* and pathogen virulence genes that involve beating the host defense under incompatible and compatible interactions. That knowledge can be successfully employed in management strategies to maintain the host genotype without being overcoming by the pathogen. In addition, an analysis of the trophic characteristics of *L. maculans* was conducted.

## 2. Results

### 2.1. Response of Rlm7 Line and Westar to Avirulent UMAvr7 and Virulent Umavr7 Isolates

**Figure 1:**
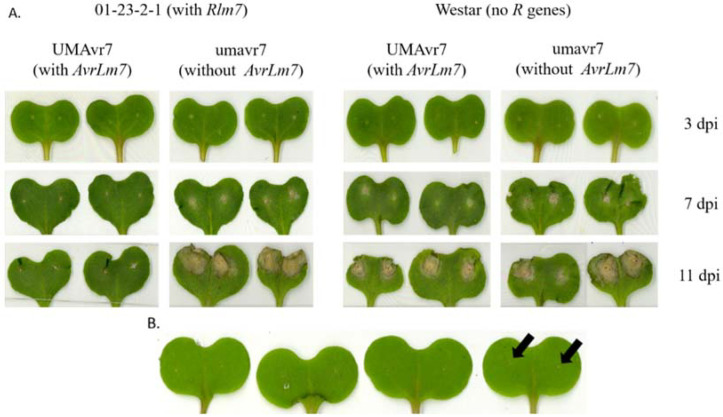
Phenotypic responses of Rlm7 line (01-23-2-1) and Westar to the infection of UMAvr7 and umavr7 at 3-, 7-, and 11-days post inoculation under controlled conditions. (**A**) UMAvr7 isolate with *AvrLm7* and umavr7 isolate without *AvrLm7* were inoculated to the Rlm7 line and Westar. Necrotic lesion development at 3-, 7-, and 11-dpi are shown. (**B**) Mock experiment. The arrows indicate the inoculated sites.

The Rlm7 line (01-23-2-1) showed no disease lesion development with the infection of UMAvr7, even after 11-dpi, except for a thin layer of dead tissue surrounding the inoculated sites. On the other hand, lesion development was observed in plants of the Rlm7 line inoculated with isolate umavr7. Westar showed lesion development with both isolates. Lesions started to show up from 7-dpi onwards among the days considered (Figure 1A). Mock samples showed only the marks of pinched sites of cotyledons (Figure 1B).

### 2.2. Transcriptomic Study Results of Dual RNA-Seq

**Figure 2:**
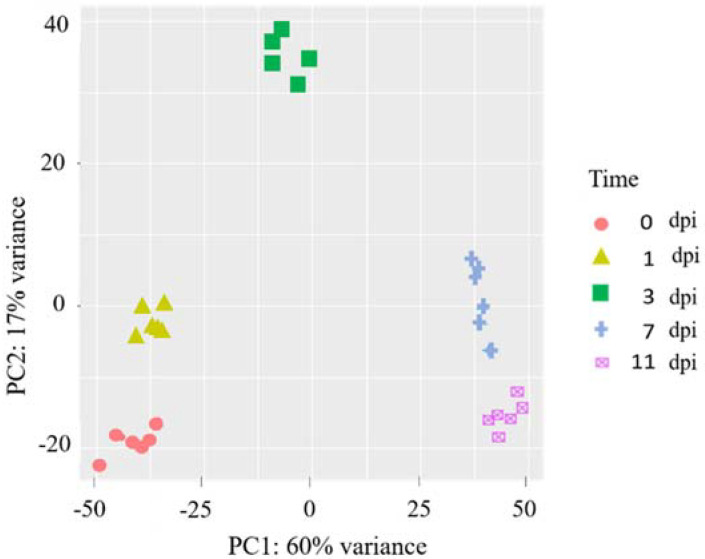
Principal components (PC) of RNA-seq expression profile obtained for *Leptosphaeria maculans* in planta. PC analysis shows RNA-seq expression profile of both UMAvr7 (with *AvrLm7*) and umavr7 (mutant of *AvrLm7*) of *L. maculans* in in planta samples of both hosts: 01-23-2-1 (Rlm7 line) and Westar (no *R* genes) at 0-, 1-, 3-, 7-, and 11-days post inoculation. The colors used were the same for both isolates at each time point. *x*-axis, PC1; *y*-axis, PC2. The proportion of variance for each PC is indicated in axis titles. Dots in the same color are three replicates of each isolate for each time point. Results were representative of three biological replicates.

The dual deep RNA-seq approach produced approximately 3,427 million raw reads generated from a total of 72 pooled samples and average of 48 million reads were obtained per single sample (Figure 2).

Principal component analysis was used to examine the characteristics of transcriptomic expressions of two *L. maculans* isolates-UMAvr7 and umavr7-within the host plant system at five different time points were considered (Figure 2). The expression profiles of biological replicates were distinctly clustered for each isolate at each timepoint. Though, samples at each timepoint exhibited clear separation, replicates of timepoints 0- and 1-dpi were clustered at one end while replicates of 7- and 11-dpi were clustered at the other end. The cluster for 3-dpi was located at another end as shown in Figure 2.

### 2.3. Expression of Avirulence Genes of L. Maculans in Planta

**Figure 3:**
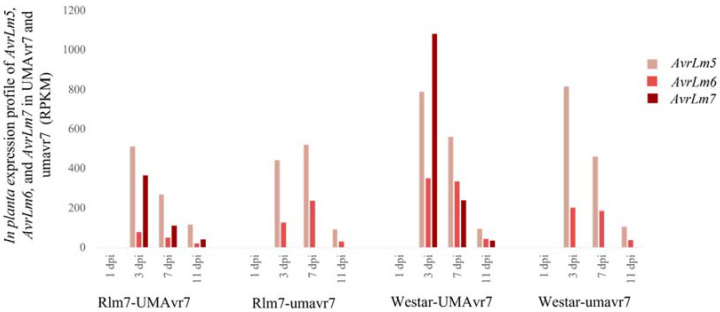
In planta expression of *AvrLm5*, *AvrLm6*, and *AvrLm7* of *Leptosphaeria maculans* isolates UMAvr7 and umavr7 in infected host plants. Dual RNA-seq results exhibited in planta expression of *AvrLm5*, *AvrLm6*, and *AvrLm7* in UMAvr7 and umavr7 isolates in both infected 01-23-2-1 (Rlm7 line) and Westar plants. The expression of *AvrLm5* and *AvrLm6* were observed in both isolates, while the expression of *AvrLm7* was shown only in UMAvr7 isolate in both hosts. Results were representative of three biological replicates.

In planta avirulence gene *AvrLm7* (Lema_P086290) expression was observed only in UMAvr7 isolate but not in umavr7 at any time point with any of the hosts (Figure 3). In planta expression of *AvrLm7* of UMAvr7 was the highest at 3-dpi with both Rlm7 line and Westar. The expression was the least at 1-dpi and the expression at 7- and 11-dpi were at intermediate levels. The expression of *AvrLm7* was comparatively higher in Westar than in Rlm7 line. In axenic cultures, both isolates did not express *AvrLm7* nor *AvrLm6* (Lema_P049940). *AvrLm5* (Lema_P070880) was observed at a low expression level (data not shown).

### 2.4. Differentially Expressed Genes at 1-, 3-, 7-, and 11-dpi

UMAvr7 showed 114 differentially expressed genes (DEGs) compared to umavr7 in axenic culture. The expression log absolute values of fold-change cutoff (logFC) ≥ 2 and false discovery rate (FDR) ≤ 0.05 were considered (Supplementary Table S1).

**Figure 4:**
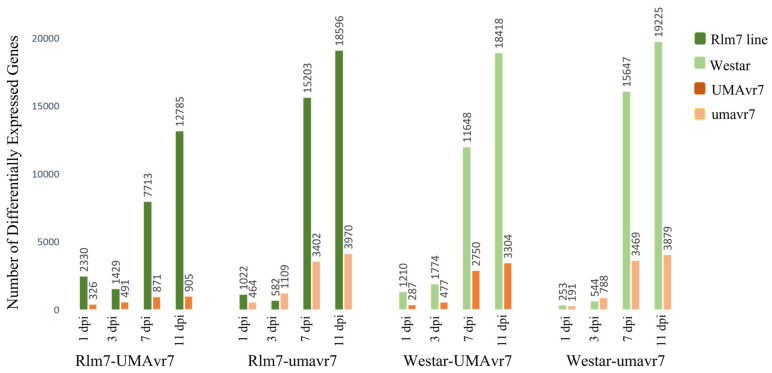
Differentially expressed genes (DEGs) in two canola genotypes and two *L. maculans* isolates under four different host-pathogen interactions. Number of DEGs in two hosts, 01-23-2-1 (Rlm7 line) and Westar (no *R* genes), and in two *L. maculans* isolates UMAvr7 (with *AvrLm7*) and umavr7 (mutant of *AvrLm7*) under each host-pathogen interaction at 1-, 3-, 7-, and 11-days post inoculation compared to 0-dpi analyzed by dual RNA-seq are shown. Results were representative of three biological replicates. The results were considered at the fold-change cutoff (logFC) of ≥1 and ≤−1 and false discovery rate (FDR) of ≤0.05.

**Figure 5:**
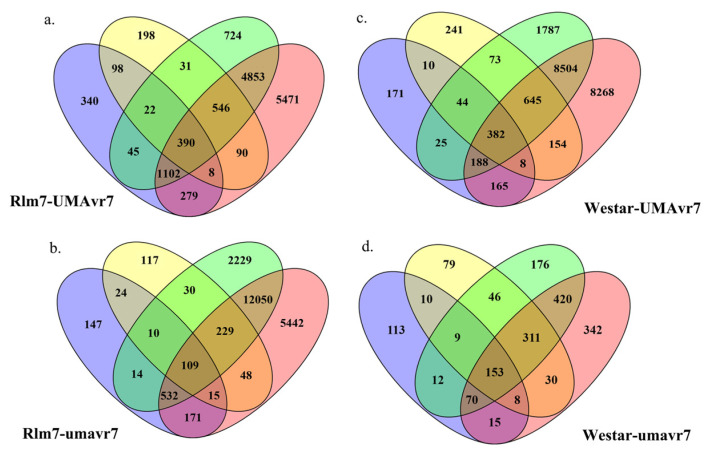

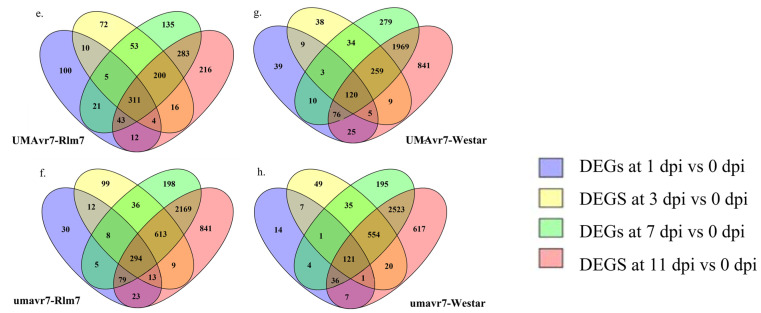
Unique and shared differentially expressed genes (DEGs) in two canola genotypes and two *L. maculans* isolates under four different host–pathogen interactions. Unique and shared DEGs in two hosts, 01-23-2-1 (Rlm7 line) and Westar, and in two *L. maculans* isolates UMAvr7 (with *AvrLm7*) and umavr7 (mutant of *AvrLm7*) under each host–pathogen interaction at 1-, 3-, 7-, and 11- days post inoculation compared to 0-dpi analyzed by dual RNA-seq. Results are representative of three biological replicates. The results were considered at the fold-change cutoff (logFC) of ≥1 and ≤−1 and false discovery rate (FDR) of ≤0.05. Unique and shared DEGs in Rlm7 line (**a**,**b**), Westar (**c**,**d**), UMAvr7 (**e**,**f**), and umavr7 (**g**,**h**) are shown incompatible and compatible interactions, respectively.

**Figure 6:**
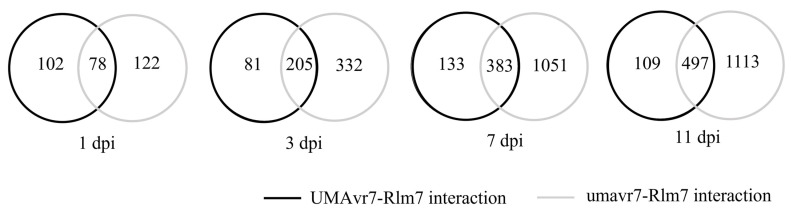
Unique and shared differentially expressed genes (DEGs) in two *Leptosphaeria maculans* isolates in interaction with 01-23-2-1 (Rlm7 line). Unique and shared DEGs in two *L. maculans* isolates UMAvr7 (with *AvrLm7*) and umavr7 (mutant of *AvrLm7*) in incompatible and compatible interactions with Rlm7 line at 1-, 3-, 7-, and 11-days post inoculation compared to 0-dpi analyzed by dual RNA-seq. Results were representative of three biological replicates. The results were considered at the fold-change cutoff (logFC) of ≥1 and ≤−1 and false discovery rate (FDR) of ≤0.05.

The DEGs of two hosts and two isolates in all four host–pathogen interactions at 1-, 3-, 7-, and 11-dpi were assessed compared to the initial time point 0dpi. Rlm7 line showed DEGs of 7713 and 12,785 at 7- and 11-dpi in incompatible interaction, and 15,203 and 18,596 in compatible interaction which is nearly two-times higher (*p* < 0.05). Pathogen isolates UMAvr7 demonstrated 326, 491, 871, 9050 number of DEGs at 1-, 3-, 7-, and 11-dpi, whereas umavr7 isolate exhibited 464, 1109, 3402, and 3970 number of DEGs in interactions with the Rlm7 line (*p* < 0.05). The expression patterns of DEGs were similar in all three compatible interactions (Figure 4). Unique and shared DEGs in two canola genotypes and two *L. maculans* isolates under four different host–pathogen interactions were analyzed as shown in Figure 5. The analysis of unique and shared DEGs in two *L. maculans* isolates in interaction with Rlm7 line is shown in Figure 6. The DEGs at 1-, 3-, 7-, and 11-dpi were higher in umavr7 isolate, which makes a compatible interaction with the Rlm7 line compared to UMAvr7 which makes an incompatible interaction (Figure 4, Figure 5 and Figure 6). The difference increased with the time as clearly shown in Figure 6.

### 2.5. Differentially Expressed Virulence Genes in the Pathogen Isolates

**Figure 7:**
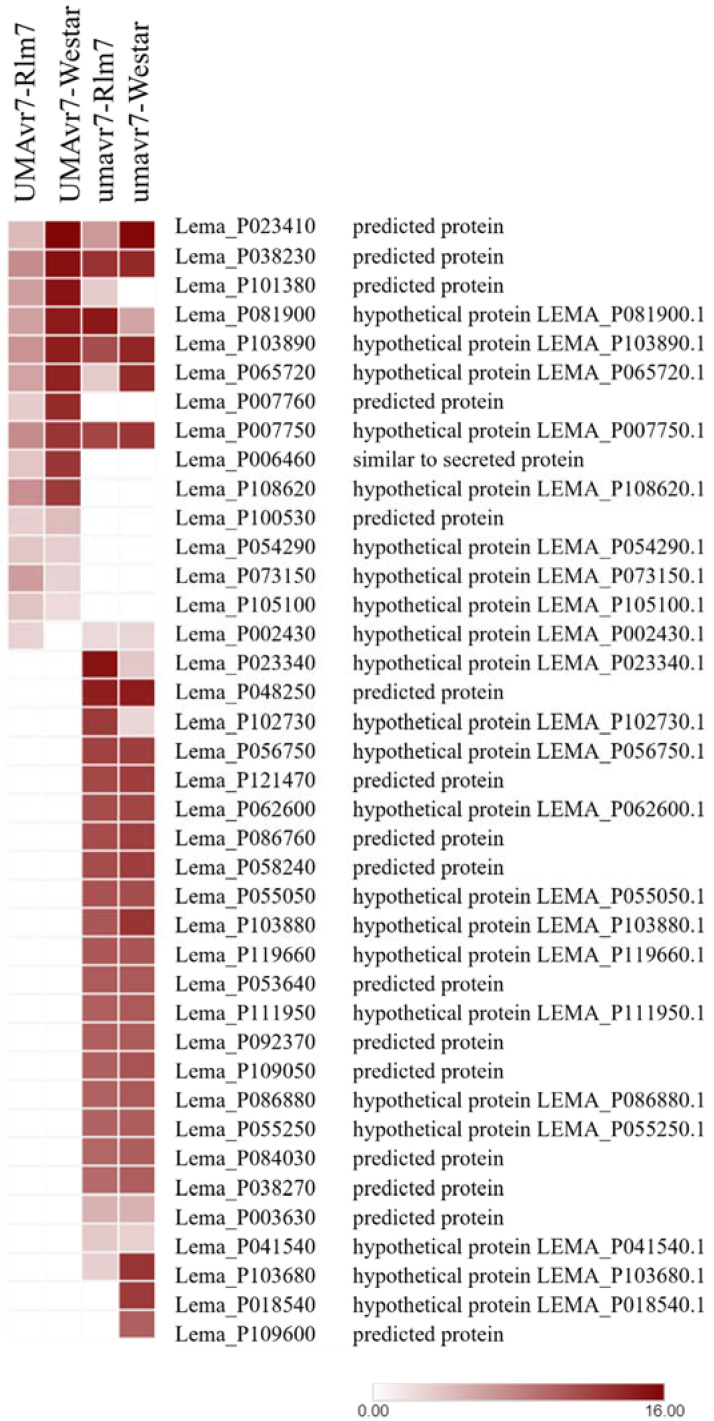
Differentially expressed effector genes of *Leptosphaeria maculans* isolates, UMAvr7 and umavr7 in *Brassica napus* genotypes, Rlm7 and Westar. The heatmap shows the differentially expressed effector genes of UMAvr7 and umavr7 in planta compared to 0-dpi. The results were considered at the fold-change cutoff (logFC) of ≥1 and ≤−1 and false discovery rate (FDR) of ≤0.05. Locus and putative annotation of *L. maculans* effector genes, and log fold-change expression of each gene under each interaction are shown. UMAvr7: wild type isolate with *AvrLm7*; umavr7: mutant of *AvrLm7*; Rlm7: *Brassica napus* genotype 01-23-2-1 with *Rlm7*; Westar: susceptible check with no *R* genes. Results were representative of three biological replicates.

In addition to the *AvrLm7* gene, there were number of other effector genes which were differentially expressed at different timepoints in both isolates in the interactions with Rlm7 line (Figure 7) and Westar (data not shown). The upregulation of effector genes in three compatible interactions were obvious compared to the incompatible interaction. There was a list of necrotrophic effectors which were expressed only at 11-dpi.

**Figure 8:**
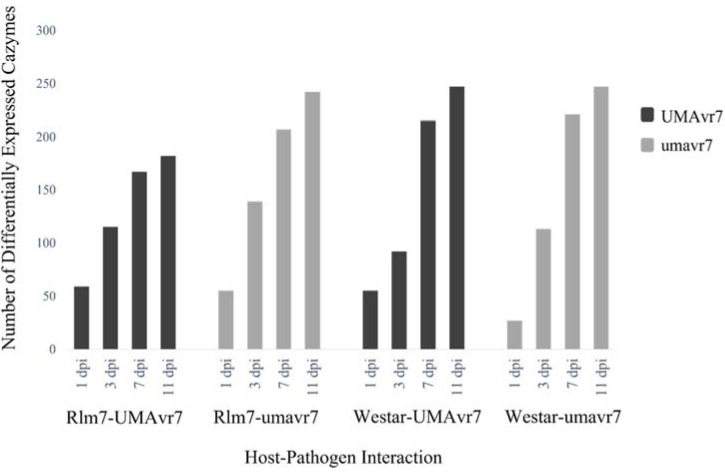
Differentially expressed CAZymes of *Leptosphaeria maculans* UMAvr7 and umavr7 isolates in infected host plants. Dual RNA-seq results exhibited differentially expressed CAZymes of UMAvr7 and umavr7 isolates in both infected 01-23-2-1 (Rlm7 line) and Westar plants. The differential expression of CAZymes was higher in compatible interactions than in the incompatible interaction. The expression level was increased with time from 1- to 11-dpi in each interaction. Results are representative of three biological replicates. The results were considered at the fold-change cutoff (logFC) of ≥1 and ≤−1 and false discovery rate (FDR) of ≤0.05.

A high number of genes encoding carbohydrate-active enzymes (CAZymes) of UMAvr7 and umavr7 were differentially expressed at latter timepoints, 7- and 11-dpi. The expressions were higher in compatible interactions compared to those in the incompatible interaction (Figure 8 and Supplementary Figure S1).

**Figure 9:**
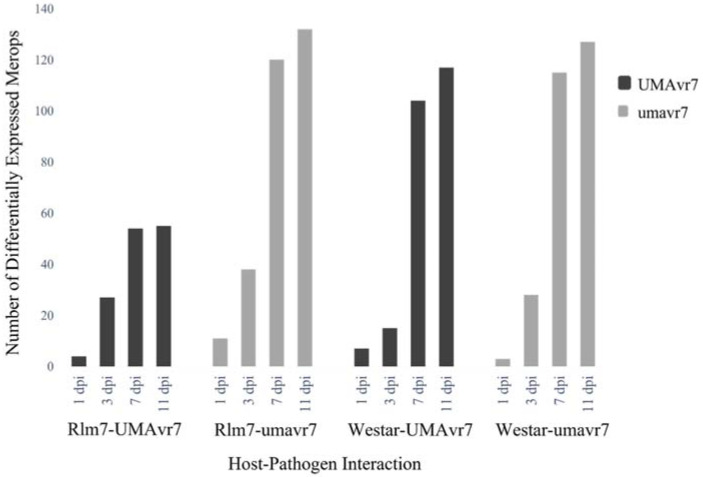
Differentially expressed Merops of *Leptosphaeria maculans* UMAvr7 and umavr7 isolates in infected host plants. Dual RNA-seq results exhibited differentially expressed Merops (peptidases) of UMAvr7 and umavr7 isolates in both infected 01-23-2-1 (Rlm7 line) and Westar plants. The differential expression of peptidases was higher in compatible interactions than in the incompatible interaction. The expression level was increased with time from 1- to 11-dpi in each interaction. Results were representative of three biological replicates. The results were considered at the fold-change cutoff (logFC) of ≥1 and ≤−1 and false discovery rate (FDR) of ≤0.05.

Furthermore, the differentially expressed Merops—which are peptidases in both isolates—were higher in compatible interactions compared to the incompatible interaction (Figure 9).

### 2.6. Differentially Expressed Defense-Related Genes in the Host Plants

**Figure 10:**
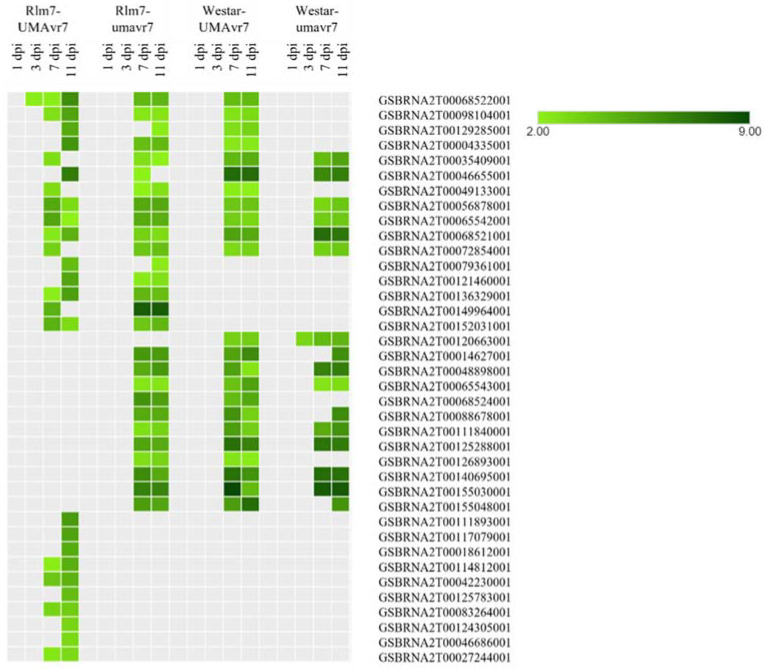
Differential expression of NLR genes of host plants. Differential expression of NLR gene in Rlm7 and Westar plants under Rlm7–umavr7, Rlm7–umavr7, Westar-UMAvr7, and Westar-umaver7 interactions are shown. Logarithm of absolute value of the fold-change cutoff (logFC) was ≥2, and false discovery rate (FDR) was ≤0.05.

The differential expression of NLRs of two host plants under incompatible and compatible interactions was clearly observed at the fold-change cutoff (logFC) ≥ 2 and false discovery rate (FDR) ≤ 0.05 (Figure 10). In all three compatible interactions, the upregulation of NLRs was clearly observed from 7-dpi onwards. In contrast, upregulation of one NLR gene (GSBRNA2T00068522001) was observed at early as 3-dpi onwards in incompatible interaction. The upregulation of NLRs: GSBRNA2T00035409001, GSBRNA2T00046655001, GSBRNA2T00049133001, GSBRNA2T00056878001, GSBRNA2T00065542001, GSBRNA2T00068521001, and GSBRNA2T00072854001 were seen in all four interactions. There was a set of NLRs specifically upregulated only in the incompatible interaction (Figure 10). In addition, differential expression of RLPs and SPs were also upregulated at later parts, i.e., 7- and 11-dpi in each interaction. GSBRNA2T00025294001 was upregulated in Rlm7 plant under incompatible interaction. On the other hand, Westar demonstrated upregulation of GSBRNA2T00006438001 under its compatible interactions with both isolates (data not shown).

**Figure 11:**
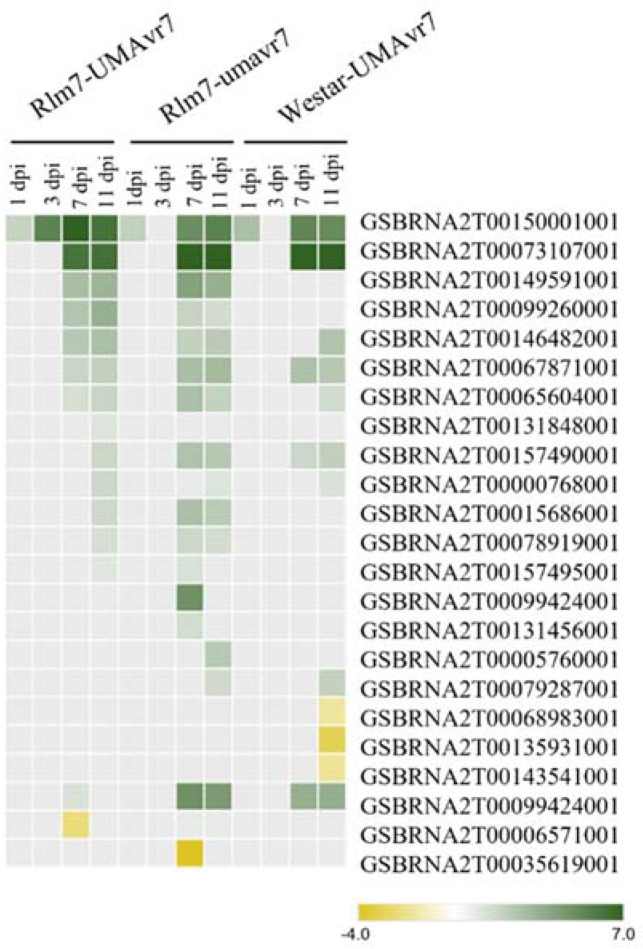
Differential expression of genes related to salicylic acid biosynthesis and signaling pathways. Rlm7-umavr7, Rlm7-umavr7, and Westar-UMAvr7 interactions are shown. Logarithm of absolute value of the fold-change cutoff (logFC) ≥ 2, and false discovery rate (FDR) ≤ 0.05 were considered.

**Figure 12:**
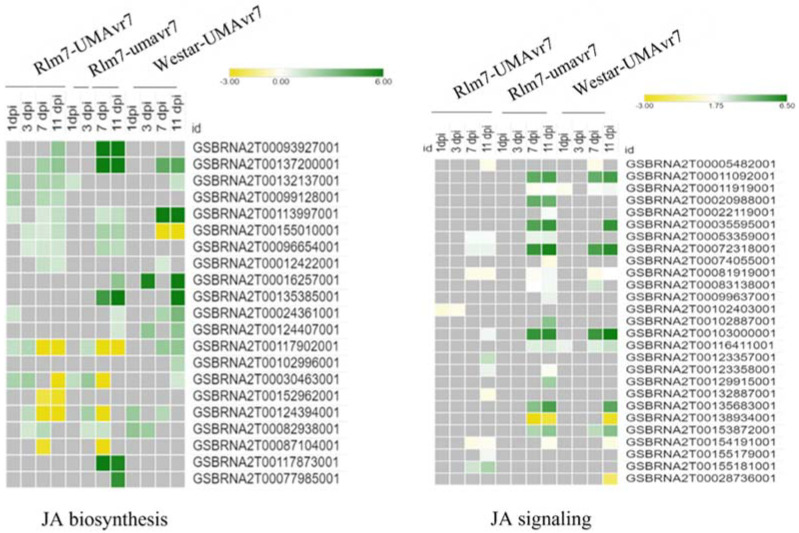
Differential expression of genes related to Jasmonic acid biosynthesis and signaling pathways. Rlm7-umavr7, Rlm7-umavr7, and Westar-UMAvr7 interactions are shown. Logarithm of absolute value of the fold-change cutoff (logFC) ≥ 2, and false discovery rate (FDR) ≤ 0.05.

Differentially expressed genes related to SA biosynthesis and signaling in the host genotypes was observed at 1-dpi in all three interactions considered. However, *PR1* (GSBRNA2T00150001001) gene upregulation was demonstrated in the incompatible interaction at 3-dpi, which was absent in other two compatible interactions shown. The genes related to SA biosynthesis and signaling were highly expressed in all three interactions at later stages as shown in Figure 11. A higher number of genes related to JA biosynthesis and signaling pathway were differentially expressed starting earlier (1- and 3-dpi) in the incompatible interaction compared to compatible interactions. However, copatible interactions demonstrated higher upregulation of those genes compared to the incompatible interaction (Figure 12).

### 2.7. RNA-seq Data Validation by RT-qPCR

**Figure 13:**
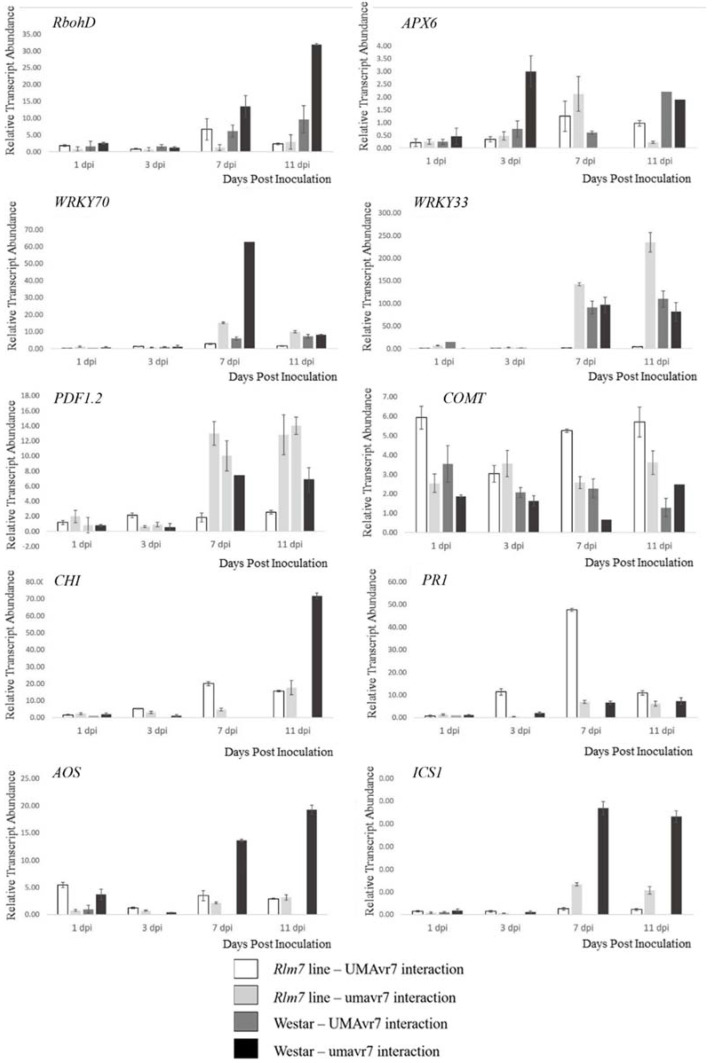
*Brassica napus* gene expressions followed by inoculation with *Leptosphaeria maculans*. Relative transcript abundance of RBOHD, APX6, WRKY70, WRKY33, PDF1.2, COMT, CHI, PR1, AOS and ICS1 in susceptible (S) and resistant (R) cotyledons assessed at 1-, 3-, 7-, and 11-dpi. The interactions of Westar with both isolates have also been analyzed. Actin (GenBank accession number AF111812.1) was used as the internal control and to normalize expression data. Relative transcript abundance is normalized relative to 0-dpi in each treatment. Error bars represent standard deviation of the mean. The results are based on three replicates in three independent experiments.

*RBOHD*, *WRKY33*, *PDF1.2*, *AOS*, and *ICS1* genes involving in ROS production, JA and SA pathways, were upregulated starting from early stages (1- and 3-dpi onwards) in the incompatible interaction. These gene expressions started later in compatible interactions and expressions were highly upregulated from 7-dpi onwards (Figure 13).

## 3. Discussion

The incompatible interaction clearly exhibited an early onset of defense-related genes, further confirming the involvement of gene-for-gene interaction [7] in the canola blackleg pathosystem. In compatible interactions, the abovementioned gene expressions commenced at later points, such as 7-dpi, proving the absence of early recognition of the pathogen.

In this study, UMAvr7 (with *AvrLm7*) and canola genotype ‘01-23-2-1’ with *Rlm7* exhibited incompatible interaction where ETI led PCD due to early recognition of avirulence protein AvrLm7 by corresponding host Rlm7 protein. The Rlm7 line showed no lesion development over time, but only a thin ring of dead tissue surrounding the wound of inoculated cotyledons (Figure 1A). That layer of dead tissue was distinct from what was seen in mock samples as shown in Figure 1B, further confirming the layer observed resulted from PCD [9]. This thin layer of dead tissue and no further lesion development confirmed that PCD prevented further colonization of the pathogen in cotyledon under incompatible interaction. In contrast, both Rlm7 line and Westar showed compatible interactions with virulent isolate, umavr7.

Though the Rlm7–umavr7 interaction was compatible, the lesion development became evident only from 7-dpi. The observations explain the hemibiotrophic nature of the pathogen, which initially survives as a biotroph in the cotyledon apoplast without making any symptoms, then switching into its necrotrophic phase later in the lifecycle. There was no activation of ETI in this system due to the absence of initial recognition of pathogen avirulence protein by the host. Therefore, no activation of PCD to limit the pathogen spread throughout the cotyledon. After colonization within the cotyledon apoplast, the pathogen transferred into its necrotrophic stage, making necrotic lesions on cotyledons. The number of pathogen DEGs in incompatible and compatible interactions clearly expressed the higher numbers in compatible interactions, verifying that the pathogen was under control in the incompatible interaction.

Pathogen avirulence proteins are effectors that manipulate host cell structure and function, thereby facilitating infection of plant pathogens [43]. Transcriptomic results confirmed that umavr7 isolate was a knockout mutant by not expressing the *AvrLm7* in either of the hosts at any timepoint used (Figure 3) [49]. The higher expressions of *AvrLm7*, *AvrLm6*, and *AvrLm5* at 3-dpi confirmed the higher avirulence gene expression with the biotrophic pathogens [51]. The *AvrLm7* gene expression was lower at the necrotrophic stage. The expression was comparatively higher in Westar than that in the Rlm7 line. This observation can be explained by the case that, in Westar, the pathogen was in an environment with no *R* genes. Therefore, no barriers were exerted at the initial stage from the host due to lack of early recognition of the pathogen. Once the host defense mechanisms are activated against the pathogen at the latter stage, the pathogen would now have colonized, established, and become robust to overcome the plant defense as it was too late to be defended against. Low expression of all three avirulence genes at 1-dpi could be explained in that the pathogen was not established yet in planta at that time point. In axenic cultures, even UMAvr7 did not exhibit the expression of the gene *AvrLm7* nor *AvrLm6*, and exhibited shallow expression of *AvrLm5* (data not shown). The performances explained above indicate that *AvrLm7* was not a housekeeping gene, and it commences its expression during the infection procedure within a host plant. In addition, the results further confirmed umavr7 is a knockout mutant by not expressing the *AvrLm7* at any of the time points considered. Though Zou et al. (2020) generated the umavr7 by knocking out only the *AvrLm7* gene by CRISPR/Cas9 genome editing system and confirmed the mutant does not have any off targets in the genome by subsequent sequencing studies, UMAvr7 showed 114 DEGs compared to umavr7 under in vitro conditions (Supplementary Table S1). This observation could be explained in that the *AvrLm7* gene could be interconnected with the expression of other genes in the pathogen metabolism. Therefore, the *AvrLm7* gene might have other important roles as well in the pathogen system except its role as an effector protein.

There were a number of effector genes differentially expressed at 3-, 7-, and 11-dpi compared to 0-dpi other than *AvrLm5*, *AvrLm6*, and *AvrLm7* in isolates in the interactions with the hosts (Supplementary Figure S1). Effectors involve the reprogramming of host plant cells and modulating of plant immunity to facilitate the infection by overcoming PTI [52]. The presence of highly expressed different effector proteins in umavr7 at 7- and 11-dpi in the compatible interaction with the Rlm7 line explains the involvement of different effector proteins in the pathogenicity after inoculation. In addition, both isolates exhibited several effector genes differentially expressed only at 11-dpi, i.e., in the necrotrophic stage of the pathogen (The fold-change cutoff (logFC) ≥ 1 and ≤−1 and false discovery rate (FDR) ≤ 0.05). Necrotrophs use an arsenal of effectors to disable susceptible hosts prior to colonization [53]. They explained that necrotrophic effector function acts in an ‘inverse’ manner of gene-for-gene interaction. The interaction between a necrotrophic effector and corresponding protein produced by a host dominant sensitivity gene causes the disease. All the necrotrophic effectors shown in Table 1 are simple sequence repeats [54]. The necrotrophic effectors demonstrated comparatively low expressions in UMAvr7 isolate in its incompatible interaction with the Rlm7 line, confirming the pathogen was under control in that interaction.

The genes encoding CAZymes are important in modifying fungal cell walls and degrading plant cell walls [55]. Enzymes such as cellulases, hemicellulases, and pectinases are prominent in degrading host cell walls. Although, the majority of CAZymes were highly and differentially expressed during the necrotrophic phase (Supplementary Figure S3). According to O’Connell et al. (2012), pectin degrading enzymes become upregulated to facilitate the pathogen entering the plant system. In this study, this might not occur in the real situation as pathogen inoculation was facilitated by wounding the cotyledons. However, there were still differentially expressed CAZymes as they were required for the pathogen for getting into the host system. Differentially expressed CAZymes were higher in compatible interactions, where the pathogen colonizes and infects the host successfully compared to the resistant incompatible interaction in which the pathogen was controlled by the host.

Moreover, peptidases (also termed proteases, proteinases, and proteolytic enzymes) were highly expressed in compatible interactions compared to the incompatible interaction. Upon host penetration, fungal pathogens use proteases that degrade plant antimicrobial proteins, such as pathogenesis-related proteins, including plant chitinases and protease inhibitors that support plant immunity [56]. Generally, hemibiotrophs and necrotrophs exhibit a higher number of secreted proteases than biotrophs [57]. Jashni et al. (2015) explained that in incompatible interaction, the host plant activates its proteases and protease inhibitors, modifying or damaging pathogen proteases. The study results demonstrated lower expression of peptidases (also termed proteases, proteinases, and proteolytic enzymes) in the incompatible interaction, and their higher expressions in compatible interactions confirm the pathogen control by the host under the incompatible interaction.

Host gene expressions related to plant defense exhibited differences between compatible and incompatible interactions. With logFC ≥ 2 as a cut-off point, an upregulated NLR gene (BnaA04g11170D protein [Source: UniProtKB/TrEMBL; Acc: A0A078HQ40]) was observed as early as 3-dpi only in the incompatible interaction, suggesting the possibility of the gene being involved in gene-for-gene interaction.

In hemibiotrophic pathogens, expression of phytohormones—especially SA, JA, and ET signaling—vary between incompatible and compatible interactions [58]. As Chowdury et al. (2017) also revealed, the most exciting observation regarding host–hemibiotroph interaction was that the host plant could tune its defense strategy accordingly as the pathogen changes its trophic state. Tissues at and distal to the inoculation site exhibited accumulation of defense signals including phytohormones such as SA [59,60], and subsequently systemic production of pathogen-related antimicrobial proteins (PRs) such as Pathogenesis-Related Protein 1 (PR1) [61]. As shown in Figure 9, *PR1* (GSBRNA2T00150001001) gene expression was upregulated in all three interactions at even 1-dpi around a logFC value of two. This observation could be due to the initial stress caused by the abiotic stress caused in the cotyledons due to wounding [62]. However, the differential expression of the gene was raised more than 10 times at 3-dpi in the Rlm7 line in the incompatible interaction, while all other compatible interactions did not show a differential expression of *PR1* gene at 3-dpi.

All most all NLR genes were considered as demonstrating relatively late expressions, suggesting it less likely that NLR genes are involved in regulation of the biotrophic phase of the pathogen. Interestingly, the *B. napus* gene GSBRNA2T00068522001 (BnaA04g11170D) was expressed early as 3-dpi of pathogen colonization (Figure 12) in the incompatible interaction. BnaA04g11170D as a crucial drought responsive gene [63] and a disease resistance gene [64].

The outstanding high expression of *PR1* in the incompatible interaction, Rlm7 line-UMAvr7, could prove the early recognition and activation of plant defense due to gene-for-gene interaction. Moreover, in the compatible interactions, the pathogen might be colonizing in the host apoplast without harming the plant cells during its biotrophic phase so that the host cannot identify the threat within. Subsequently, the pathogenic fungus becomes virulent once it reaches the required quorum sensing (QS). QS is a density-dependent signaling mechanism of microbes. When their signaling compounds reach the threshold level, it synchronizes the expression of virulence factors as a function of the fungal density to overcome the host defense [65]. Therefore, it could be suggested that the pathogen reached its QS and started establishing the infection so the host plant could recognize its rival. As the pathogen switches into its necrotrophic phase, the killing of host tissues for survival could be observed by lesion development. Based on the lesion sizes seen at 7-dpi, it was clear lesion development had been started between 3- and 7-dpi. Based on previous observations, it is possible to mark this point at 5- or 6-dpi [66]. Unfortunately, we did not set a timepoint within that period to confirm this.

SA plays a vital role in PTI, ETI, and systemic acquired resistance (SAR) [35,36]. SA is involved in establishing and maintaining SAR [67]. SA is also responsible for signal transduction. Reduced SA accumulation relates to lowering basal defense gene expression leading to susceptibility [64] and can explain disease development in the compatible interactions. SA impacts other hormonal pathways, such as JA and ET, through antagonistic and synergistic interactions [68]. Incompatible interaction led by ETI causes PCD to control the further spreading of biotrophs as they cannot survive on dead tissues. Interestingly, Spoel et al. (2007) suggested that ETI does not suppress JA-dependent defense, which results in the plant being protected by necrotrophs even with PCD condition as JA is the key phytohormone in defense against necrotrophs. Differential expression levels of JA and ET of the host plant under the incompatible interaction were lower than the compatible interactions. The compatible interactions exhibited clear raised expressions of JA biosynthesis, JA signaling, ET biosynthesis, and ET signaling genes at the latter part, 7- and 11-dpi, in which the pathogen survived as a necrotroph (Figure 10). JA is one of the significant phytohormones which plays a vital role in plant defense against necrotrophic pathogens [69]. Our observations can further confirm this as it did not show upregulation in gene expression related to JA in the incompatible interaction. In contrast, SA levels remain upregulated at later stages studied: 7- and 11-dpi. This continued upregulation of SA levels in Rlm7–umavr7 interaction could be due to the SAR developed in the plant system. On the other hand, it can suggest that the pathogen is under control in the system and the remaining population might last as biotrophs at later time points studied. Chowdhury et al. (2017) also explained that the biotrophy-to-necrotrophy switch was delayed in incompatible interactions.

Therefore, these results suggest that in the canola blackleg pathosystem, the host plant regulates the pathogen under incompatible interaction by limiting it at its initial infectious site, and the residual pathogen remains in a biotrophic phase without switching into the necrotrophic phase until dies without required resources. In addition, this observation verifies what we suggested regarding reaching the QS to start its mission as a necrotroph, being the stage, which is the real threat to the host. In addition, host defense-related genes—especially SA and JA biosynthesis and signaling-related genes, GSBRNA2T00150001001 (*PR1*) and GSBRNA2T00068522001 in NLR gene family—started to upregulate early in the incompatible interaction, confirming the early recognition of the pathogen. The high upregulations of the aforementioned genes after 7-dpi only in the compatible interactions confirmed the active pathogen virulence in compatible interactions, but not in the incompatible interaction in which the pathogen was under control.

The findings of this study will help the canola blackleg research and breeding programs to identify genes controlling host defense against *L. maculans* and pathogen genes related to its virulence in disease development. The thorough understanding of *Rlm7-AvrLm7* pathosystem will be significant to maintain the *Rlm7* genotype last without breaking down.

## 4. Materials and Methods

### 4.1. The Plant Material

*B. napus*, line 01-23-2-1, carrying (*Rlm7*) and Westar (no *R* genes) was used as the host germplasm. Three replicates were used for each treatment. Seeds were grown in Sunshine mix #4 (SunGro Horticulture, Canada Ltd., Vancouver, BC, Canada) in a growth chamber with a under a 16 h photoperiod (18 ℃ dark and 21 ℃ light).

### 4.2. Wild-Type L. maculans Isolate UMAvr7 and AvrLm7 Mutant Isolate Umavr7

*L. maculans*, isolate UMAvr7 (with *AvrLm5-6-7*) and *AvrLm7* mutant of the same isolate umavr7 (with eight base pair deletions in the coding region) generated by using CRISPR/Cas9 system [50] were grown on V8 juice agar (20% *(v*/*v)* V8 vegetable juice, 0.075% *(w*/*v)* CaCO_3_, 1.5% *(w*/*v)* agar, and 100 µg mL^−1^ of streptomycin) at 22 °C under room temperature. One single pycnidiospore isolated from each culture was used to obtain pure cultures of each isolate for further steps. After sporulation, pycnidiospores were collected in sterilized dH_2_O.

Pycnidiospore suspensions were diluted to a final concentration of 2 × 10^7^ spore mL^−1^. Seven-day-old seedlings of Rlm7 line and Westar were punctured with a modified tweezer for one wound per each lobe and then were inoculated with a 10 μL droplet of inoculum (four inoculation sites per seedling). Both host genotypes were inoculated with UMAvr7 and umavr7). Inoculated cotyledons were air dried for at least 12 h before watering. The mock control seedlings were treated the same, but with sterilized dH_2_O instead of the inoculum.

### 4.3. Sample Preparation for RNA-Seq

*B. napus* cotyledons were collected at 0, 1-, 3-, 7-, and 11-days post inoculation (dpi) with three biological replicates from each treatment at each time point. Then the samples were frozen with liquid nitrogen and were stored at −80 °C. RNA was extracted using 100 mg of ground tissue using RNeasy Plant Mini Kit (Qiagen Cat. No. 74904, Valencia, CA, USA) according to the manufacturer’s instructions. Three biological samples from each treatment at each time point were used. The quantity and quality of each sample were analyzed by using NanoDrop ND-1000 spectrophotometer (Thermo Scientific, Waltham, MA, USA) and the 2100 Bioanalyzer (Serial No. DE13806078, Agilent Technologies, Palo Alto, CA, USA). RNA integrity and purity were analyzed further by running in 2% agarose electrophoresis gel. RNA samples (samples with RIN > 7) were sent to Genome Quebec, Quebec, Canada for RNA-seq library preparation and sequencing. The sequencing was conducted using the Illumina 4000 HiSeq PE100 method.

### 4.4. RNA-Seq Analysis

The quality of RNA-seq reads was checked with Fastqc. Read processing by clipping of barcode adapters from RNA-seq reads and removing of low-quality reads were conducted (read quality < 30) using Trimmomatic software [70]. Reads were trimmed from both ends until the average of all 5-bp sliding windows reached a Phred score of 25 or higher and all of the sequences shorter than 30 bases were discarded. Processed quality reads were aligned to the *L. maculans* (https://fungi.ensembl.org/Leptosphaeria_maculans/Info/Annotation/, accessed on 1 September 2015) [71] and *B. napus* (https://www.genoscope.cns.fr/brassicanapus/, accessed on 15 October 2015) [72] genomes and transcriptomes with Tophat2 [73]. Two-base mismatches were allowed for the alignment. The minimum and maximum intron length were set to 50 and 500,000, respectively. Reads aligned to multiple sites were removed prior to further analysis.

Identification of unannotated transcripts was carried out using Cufflink tools. The gene expression level for each annotated and unannotated transcript were estimated as the number of fragments (reads) per kilobase of transcript per million mapped reads (FPKM). HTseq tool was used to count reads. The differentially expressed genes (DEGs) were identified using Omicsbox software (https://www.biobam.com/omicsbox, accessed on 5 May 2019) [74]. Genes were considered as significantly differentially expressed with FDR < 0.05 and the absolute value of log fold-change >2 and <−2 except when otherwise mentioned. The quartile normalization that excludes the top 25% of expressed genes to improve detection of less abundant genes. The -M option to mask rRNA, -b, and -u options for bias correction and to normalize for aligned tags instead of total tags were used with the Cufflink software. HTseq tool was used to count reads prior to DEGs identification. Sample quality and expression levels of transcripts were estimated using the DESeq2 package [75].

### 4.5. RNA-Seq Data Validation by RT-qPCR

Frozen cotyledons (collected at 0-, 1-, 3-, 7-, and 11-dpi) were ground in liquid nitrogen with a sterilized pestle and mortar. Total RNA was extracted using PureLink^®^ Plant RNA Reagent (Invitrogen, Carlsbad, CA, USA) and treated with a TURBO DNA-free^TM^ Kit (Ambion, Austin, TX, USA) according to the manufacturer’s instructions. Reverse transcription of the first-strand cDNA was performed using the 1st-Strand cDNA Synthesis Kit (Thermo Scientific, Waltham, MA, USA) with 1 μg of total RNA. Next, 4.2 μL of 100-fold diluted cDNA, 5 μL of PowerUp™ SYBR™ Green Master Mix (Clontech, Palo Alto, CA, USA), and 0.4 μL of each primer (10 mM) were used for PCR reaction. Primer sequences are shown in Table 2. Real time quantitative PCRs were performed on a CFX96 Real-Time Instrument (Bio-Rad, USA) with the amplification program of 50 °C for 2 min, 95 °C for 2 min, 40 cycles of 95 °C for 15 s, 60 °C for 1 min. Melting curve analysis was performed by increasing 0.5 °C at 5 s/step from 65 to 95 °C. The relative gene expression level was calculated using the 2^−ΔΔCT^ method [76].

## Figures and Tables

**Table 1:** *Leptosphaeria maculans* effector genes differentially expressed only at 11-dpi (necrotrophic stage) compared to 0-dpi. Name of effector gene, and log fold-change expression of each gene under each interaction are shown. Results are representative of three biological replicates. The results were considered at the fold-change cutoff (logFC) of ≥1 and ≤−1 and false discovery rate (FDR) of ≤0.05. UMAvr7: wild type isolate with *AvrLm7*; umavr7: mutant of *AvrLm7*; Rlm7: *Brassica napus* genotype 01-23-2-1 with *Rlm7;* Westar: susceptible check with no *R* genes.

***Leptosphaeria maculans* locus**	**Gene Name**	**UMAvr7**		**Umavr7**	
		**Rlm7**	**Westar**	**Rlm7**	**Westar**
Lema_P007750	hypothetical protein LEMA_P007750.1	7.32	12.81	11.79	12.74
Lema_P038230	predicted protein	7.22	15.15	13.09	13.66
Lema_P108620	hypothetical protein LEMA_P108620.1	7.04	12.44		
Lema_P103890	hypothetical protein LEMA_P103890.1	6.87	14.31	11.24	13.83
Lema_P073150	hypothetical protein LEMA_P073150.1	6.17	2.95		
Lema_P101380	predicted protein	6.07	14.91	3.31	
Lema_P081900	hypothetical protein LEMA_P081900.1	6.00	14.57	14.69	5.72
Lema_P065720	hypothetical protein LEMA_P065720.1	5.83	14.05	3.36	13.46
Lema_P023410	predicted protein	4.38	15.83	6.47	15.77
Lema_P105100	hypothetical protein LEMA_P105100.1	3.76	2.31		
Lema_P006460	similar to secreted protein	3.66	12.77		
Lema_P054290	hypothetical protein LEMA_P054290.1	3.65	3.11		
Lema_P007760	predicted protein	3.23	13.47		
Lema_P100530	predicted protein	3.02	4.28		
Lema_P048250	predicted protein			14.24	14.39
Lema_P103680	hypothetical protein LEMA_P103680.1			3.07	12.91
Lema_P103880	hypothetical protein LEMA_P103880.1			10.73	12.90
Lema_P018540	hypothetical protein LEMA_P018540.1				12.49
Lema_P056750	hypothetical protein LEMA_P056750.1			11.99	12.26
Lema_P121470	predicted protein			11.67	12.25
Lema_P058240	predicted protein			11.27	12.23
Lema_P086760	predicted protein			11.34	12.15
Lema_P062600	hypothetical protein LEMA_P062600.1			11.37	11.83
Lema_P055050	hypothetical protein LEMA_P055050.1			10.96	11.30
Lema_P109050	predicted protein			10.11	10.94
Lema_P119660	hypothetical protein LEMA_P119660.1			10.59	10.74
Lema_P086880	hypothetical protein LEMA_P086880.1			10.05	10.53
Lema_P111950	hypothetical protein LEMA_P111950.1			10.17	10.51
Lema_P053640	predicted protein			10.39	10.51
Lema_P092370	predicted protein			10.17	10.35
Lema_P055250	hypothetical protein LEMA_P055250.1			9.90	10.30
Lema_P084030	predicted protein			9.74	10.29
Lema_P038270	predicted protein			9.38	10.25
Lema_P003630	predicted protein			4.89	4.91
Lema_P023340	hypothetical protein LEMA_P023340.1			15.05	3.61
Lema_P079720	similar to female reproductive tract protease GLEANR_897			13.95	3.47
Lema_P102730	hypothetical protein LEMA_P102730.1			12.58	2.60

**Table 2:** List of selected defense-related genes in *Brassica napus* and their forward and reverse primers used for RT-qPCR.

Gene	Full Name	Defense Signaling Pathway	Forward Primer (5′3′)	Reverse Primer (5′3′)
WRKY70	WRKY transcription factor 70	SA signaling pathway	ACATACATAGGAAACCACACG	ACTTGGACTATCTTCAGAA TGC
PR1	Pathogenesis-related protein 1	SA pathway	GGCTAACTATAACCACGATTC	GTTCCACCATTGTTACACC
WRKY33	WRKY transcription factor 33	JA signaling pathway	TGTCGGACAGCTTGGGAAAG	AGAGGACGGTTACAACTG GAGAAA
PDF1.2	Plant defensin 1.2	Ethylene and JA pathway	AAATGCTTCCTGCGACAACG	AGTCCACGTCTCCGATCT CT
RbohD	Respiratory burst oxidase homolog protein D	ROS production	TATCCTCAAGGACATCATCAG	TATCCTCAAGGACATCATCAG
APX6	Ascorbate peroxidase	ROS scavenging	AGTTCGTAGCTGCTAAATATT	GGAGTTGTTATTACCAAGAAA
CHI	Chitinase	Pathogen chitin degradation	TGCTACATAGAAGAAATAAACGG	TTCCATGATAGTTGAATC GG
COMT	Caffeic acid O-methyltransferase	Lignin biosynthesis	TCTTCAAGAATTTCACGCAGTG	CGTCCCTAAAGGTGATGCTATT
AOS	Allene oxide synthase	Involved in JA biosynthesis	CGCCACCAAAACAA CAAA	GGGAGGAAGGAGAGAGG TTG
ICS1	Isochorismate synthase 1	Involved in SA biosynthesis	AGCGTGACTTACTAACCAG	CAAACTCATCATCTTCCCTC
Act	Actin	Reference gene	CTGGAATTGCTGACCGTATGAG	GTTGGAAAGTGCTGAGG GATG

## Data Availability

Not applicable.

## Supplementary Materials

The following supporting information can be downloaded at: https://www.mdpi.com/article/10.3390/ijms23073964/s1.
