# Supplementary material for: Leptosphaeria maculans-Brassica napus Battle: A Comparison of Incompatible vs. Compatible Interactions Using Dual RNASeq
Source: Int J Mol Sci. 2022 Apr 2;23(7):3964. doi: 10.3390/ijms23073964 (PMC8999614; doi:10.3390/ijms23073964)
Supplement: Supplementary file 1 [file ijms-23-03964-s001.zip › ijms-1635670-supplementary.pdf]

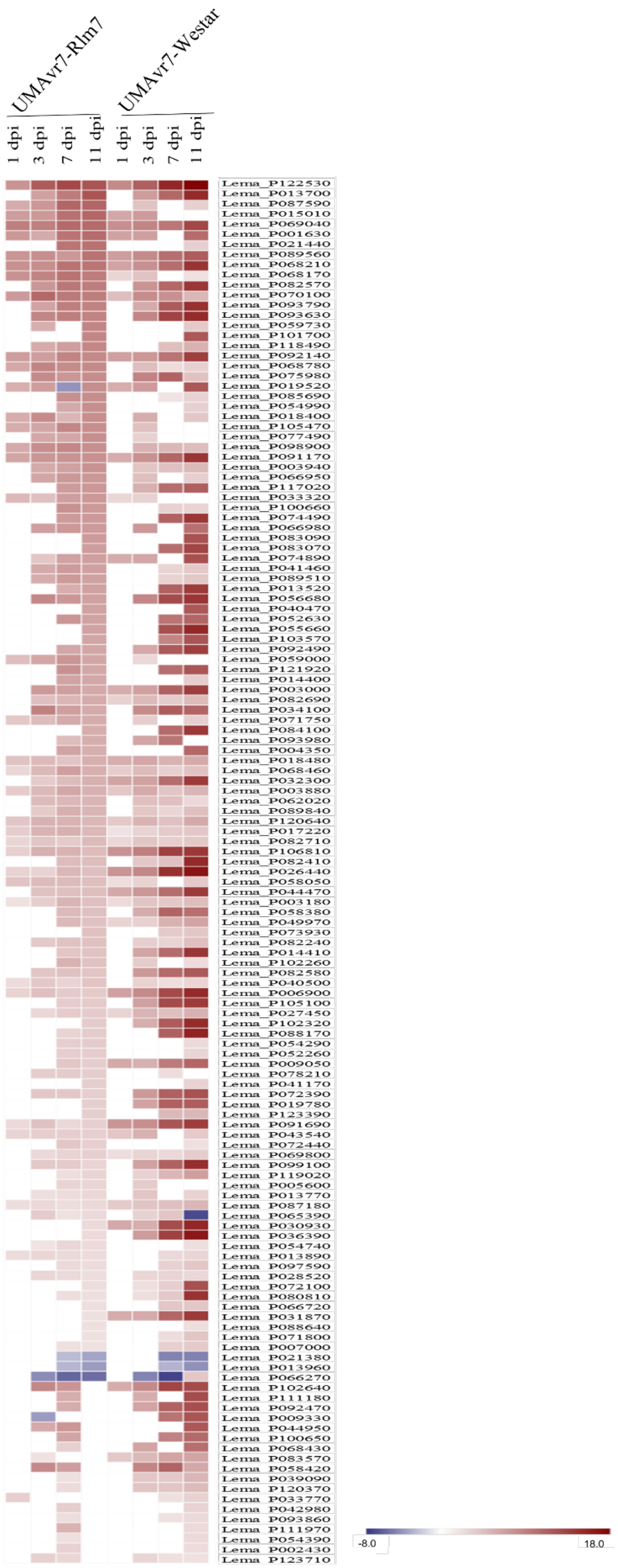

Supplementary Figure S1: Differentially expressed Cazyme genes of *Leptosphaeria maculans* isolates, UMAvr7 and umavr7 *in planta* of Rlm7. Differentially expressed Cazymes genes of

UMAvr7 and umavr7 isolates *in planta* of Rlm7 at 1, 3-, 7-, and 11- dpi compared to zero dpi. Logarithm of absolute value of fold change cutoff (logFC) ≥ 2 and ≤ -2 with false discovery rate (FDR) ≤ 0.05 were considered.

Supplementary Table S1: Differentially expressed genes (DEGs) in UMAvr7 compared to umavr7 in Axenic culture. UMAvr7 isolate showed 114 DEGs compared to umavr7 in in vitro with expression log of absolute value of fold change cutoff (logFC) ≥ 2 and false discovery rate (FDR) ≤ 0.05 were considered.

Gene locus	Log2 Fold Change	FDR p-value correction
Lema_P001940	9.51	4.0979E-295
Lema_P001950	8.90	1.5584E-111
Lema_P001960	5.86	1.1446E-169
Lema_P113300	4.86	1.41803E-98
Lema_P007210	4.84	1.9681E-113
Lema_P105290	4.71	2.2307E-142
Lema_P004820	4.67	3.05714E-53
Lema_P020960	4.50	2.94213E-88
Lema_P019630	4.20	2.43506E-73
Lema_P019640	4.14	1.17187E-77
Lema_P105280	3.96	2.95928E-55
Lema_P029920	3.93	7.1182E-198
Lema_P109850	3.93	5.4742E-129
Lema_P048740	3.92	0.049309236
Lema_P110640	3.80	6.9109E-106
Lema_P002480	3.67	2.27949E-60
Lema_P116240	3.64	1.37115E-50
Lema_P051470	3.62	8.47278E-81
Lema_P124100	3.53	2.96317E-83
Lema_P005170	3.53	0.049309236
Lema_P116170	3.53	6.17969E-55
Lema_P067150	3.49	2.91884E-75
Lema_P116460	3.42	1.02742E-51
Lema_P051480	3.34	1.325E-100
Lema_P118290	3.33	4.39695E-83

Lema_P112380	3.27	3.1833E-117
Lema_P004780	3.25	3.11884E-43
Lema_P006920	3.24	5.5316E-113
Lema_P060030	3.21	5.0406E-53
Lema_P073150	3.20	4.1134E-199
Lema_P016760	3.20	1.15427E-84
Lema_P074180	3.17	1.71756E-63
Lema_P115080	3.16	1.351E-109
Lema_P114820	3.10	2.1175E-55
Lema_P087320	3.04	1.02126E-48
Lema_P067600	3.01	3.8603E-111
Lema_P048340	3.00	1.86522E-96
Lema_P087800	3.00	1.05706E-90
Lema_P016030	2.98	3.19255E-47
Lema_P083420	2.95	6.32777E-82
Lema_P087810	2.95	1.86099E-84
Lema_P116190	2.95	8.4746E-55
Lema_P032080	2.92	6.22955E-93
Lema_P021040	2.89	2.1056E-99
Lema_P022070	2.87	3.75511E-94
Lema_P110690	2.84	9.1801E-131
Lema_P057900	2.82	1.0829E-103
Lema_P034940	2.78	2.20628E-79
Lema_P077070	2.77	7.05183E-61
Lema_P093520	2.72	1.85349E-62
Lema_P103220	2.69	8.15585E-43
Lema_P078930	2.67	8.47748E-87
Lema_P010440	2.67	9.55827E-46
Lema_P002320	2.66	2.31015E-64
Lema_P039910	2.63	0

Lema_P063620	2.55	9.2406E-128
Lema_P113070	2.49	0
Lema_P113100	2.48	0
Lema_P063060	2.48	3.2019E-101
Lema_P034930	2.47	2.3298E-71
Lema_P087610	2.45	1.29016E-93
Lema_P009910	2.45	5.4094E-107
Lema_P001850	2.44	0
Lema_P004530	2.44	2.23944E-57
Lema_P113090	2.43	0
Lema_P010470	2.42	0
Lema_P060020	2.38	1.83867E-93
Lema_P099810	2.37	4.9907E-119
Lema_P012380	2.35	3.73618E-44
Lema_P039000	2.30	2.99357E-47
Lema_P059260	2.27	1.93156E-43
Lema_P113130	2.25	0
Lema_P056470	2.25	1.13524E-42
Lema_P073380	2.25	2.34446E-81
Lema_P113120	2.23	0
Lema_P104820	2.22	2.3496E-67
Lema_P066320	2.18	9.78125E-60
Lema_P113110	2.15	0
Lema_P021830	2.12	6.94102E-46
Lema_P037530	2.06	0
Lema_P025430	2.06	0
Lema_P011200	2.05	3.1841E-89
Lema_P093680	2.03	1.15947E-62
Lema_P023990	2.02	0
Lema_P116780	2.01	1.13524E-42
